# Supplementary material for: Exploring tumor endothelial cells heterogeneity in hepatocellular carcinoma: insights from single-cell sequencing and pseudotime analysis
Source: PeerJ. 2024 Oct 28;12:e18362. doi: 10.7717/peerj.18362 (PMC11526786; doi:10.7717/peerj.18362)
Supplement: Supplemental Information 1 [file peerj-12-18362-s001.docx]

Supplementary materials

Table S1. The specific primers of genes.

| Name | Forward (5’-3’) | Reverse (5’-3’) |
| --- | --- | --- |
| JUN | AGTTCCTGTGCCCCAAGAACG | CCTGCCACCGAGGCTACC |
| PGF | GAAGTGTGGGGCCGCAGC | CTCATCGCCGCAGCAGCC |
| GAPDH | GTCTCCTCTGACTTCAACAGCG | ACCACCCTGTTGCTGTAGCCAA |
